# Insufficient sampling constrains our characterization of plant microbiomes

**DOI:** 10.1038/s41598-021-83153-9

**Published:** 2021-02-11

**Authors:** Lorinda S. Bullington, Ylva Lekberg, Beau G. Larkin

**Affiliations:** 1 MPG Ranch, Missoula, MT 59801 USA; 2 Department of Ecosystem and Conservation Sciences, University of Montana, Missoula, MT 59812 USA

**Keywords:** Bioinformatics, Microbial ecology, Molecular ecology

## Abstract

Plants host diverse microbial communities, but there is little consensus on how we sample these communities, and this has unknown consequences. Using root and leaf tissue from showy milkweed (*Asclepias speciosa*), we compared two common sampling strategies: (1) *homogenizing after subsampling *(*30 mg*), and (2) *homogenizing bulk tissue before subsampling *(*30 mg*). We targeted bacteria, arbuscular mycorrhizal (AM) fungi and non-AM fungi in roots, and foliar fungal endophytes (FFE) in leaves. We further extracted DNA from all of the leaf tissue collected to determine the extent of undersampling of FFE, and sampled FFE twice across the season using strategy one to assess temporal dynamics. All microbial groups except AM fungi differed in composition between the two sampling strategies. Community overlap increased when rare taxa were removed, but FFE and bacterial communities still differed between strategies, with largely non-overlapping communities within individual plants. Increasing the extraction mass 10 × increased FFE richness ~ 10 ×, confirming the severe undersampling indicated in the sampling comparisons. Still, seasonal patterns in FFEs were apparent, suggesting that strong drivers are identified despite severe undersampling. Our findings highlight that current sampling practices poorly characterize many microbial groups, and increased sampling intensity is necessary for increase reproducibility and to identify subtler patterns in microbial distributions.

## Introduction

The human microbiome has been thoroughly studied as an integral component of human health and performance^1–3^. Similarly, the plant microbiome is critical in understanding the health and ecology of living plants^4,5^, and their rate of decay and nutrient turnover after senescence^6^. The plant microbiome is incredibly complex and diverse; just 0.1 g of foliar plant tissue can host over 100 putative fungal species^7–9^, whereas the richness of arbuscular mycorrhizal fungi (AMF) in roots is considerably lower^10^. Knowledge of the spatial heterogeneity and overall richness of plant-associated microorganisms should inform how we sample, as this may influence our understanding of the processes that shape microbial communities and their relationships with other organisms. Currently, there is no consensus on an optimal sampling strategy needed to characterize plant-associated microbial communities. In fact, there is little technical guidance on how common sampling strategies influence our interpretation and analyses of the microbial communities we observe.

Through the use of next-generation sequencing technology, we now know that biases associated with culture-based microbiome surveys leave a large proportion of microbial diversity completely undetected^11,12^. Early departures from culture-based methods, like cloning and direct extraction of environmental DNA greatly improved our understanding of plant-associated microbial communities, but they revealed new biases suggesting that our understanding of microbial communities was still incomplete^13^. Even 454 pyrosequencing often did not provide enough high-quality sequences to adequately characterize microbial communities, resulting in sequencing effort curves that failed to reach an asymptote^14,15^. Deeper, higher quality sequencing technology now allows researchers to see a more comprehensive picture of plant-associated microbial communities than previous methods. Thus, while *sequencing* effort for individual samples is no longer a bottleneck in characterizing plant-associated microbial communities, *sampling* effort may very well be.

Many advances have been made to reduce biases when processing microbial community data^16–18^, but descriptions of sampling effort or strategy are often omitted or vague, with little or no justification of methods used. Site descriptions, including information about the developmental stage or uniformity of plants to be sampled, are often too cursory. In a recent review, Dickie et al. (2018)^19^ found that 95% of metabarcoding studies examined reported inappropriate or incomplete field or sampling methods that rendered them non-reproducible. In addition to unclear methods, many studies fail to report the robustness of their sampling efforts (e.g., through species accumulation curves that display the number of species recovered for each additional sample), despite the inherent consequences of undersampling. Indeed, undersampling of microbial communities appears to have become the rule, rather than the exception. One reason for this is that time and budget constraints limit the amount of lab work that can be performed, and often it is inappropriate or impossible to destructively sample whole plants. Most of the time researchers only sample small quantities (mg) of plant tissue representing a tiny fraction of the plant’s total biomass (often < 1%). Microbial communities observed in these samples are then used to make inferences on the larger population of colonizing microbes. In uncontrolled field studies where there are countless environmental drivers, this incomplete sampling may obscure subtle underlying patterns in microbial distributions^20^, which in turn could compromise our estimates about their diversity and the degree to which sites, treatments and individual hosts truly differ.

**Figure 1:**
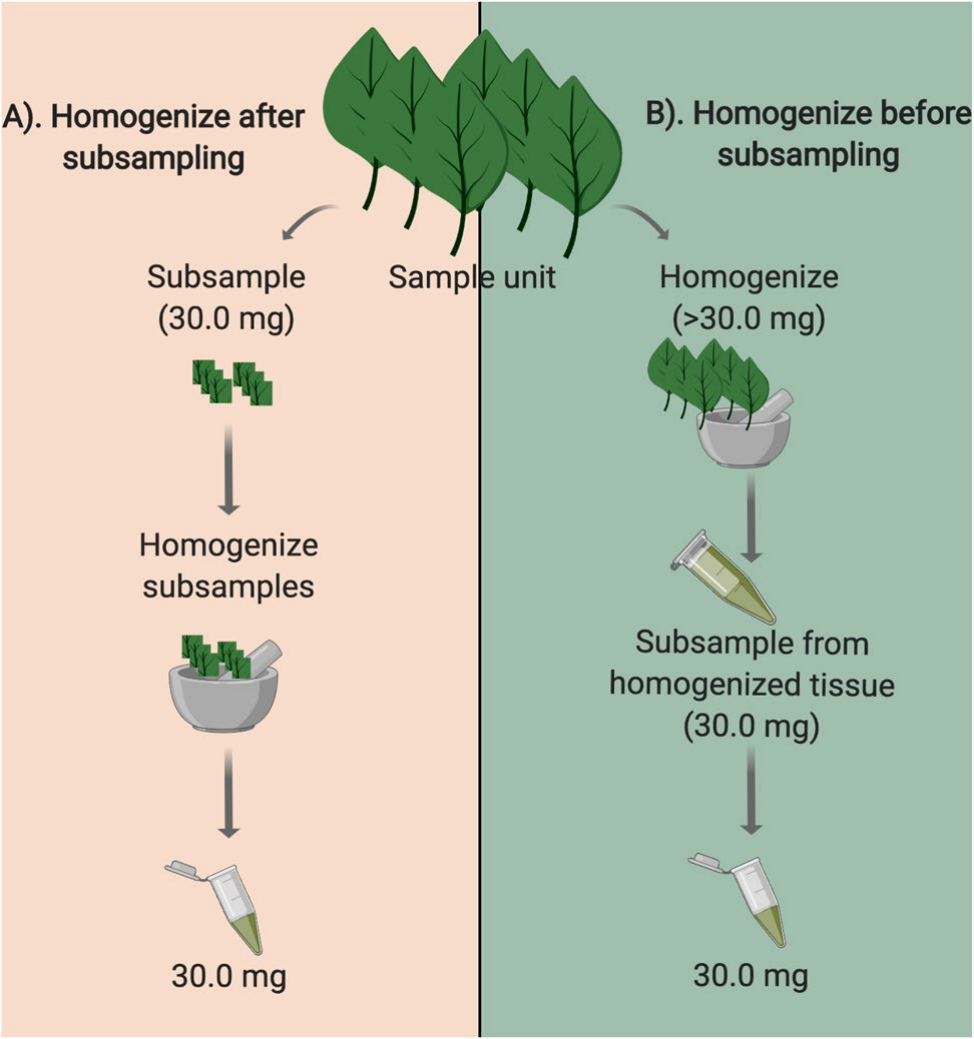
Description of the two common strategies used to subsample plant-associated microbial communities. This figure was created with BioRender.com.

Not only do we sample small proportions of total plant biomass, but researchers often differ in how these samples are collected and processed. To provide guidance for sampling plant-associated microbial groups, we compared two of the more common sampling strategies seen in the literature to see if they differ in richness and compositional estimates and if this depends on the microorganism targeted. The first sampling strategy involves collecting a specific surface area or volume of plant tissue (e.g. leaf discs or lengths of root segments) followed by tissue homogenization (e.g.,^14,21,22^). In this strategy, the spatial extent of available plant tissue is somewhat maintained, but many taxa may be missed if their distributions are patchy, which in turn may lead to increased variance in richness and composition among samples. From hereon we will refer to this strategy as “*homogenizing tissue after subsampling*” (Fig. 1A). The second common sampling strategy involves either collecting a pre-specified amount of tissue from each plant (e.g., six leaves or root segments per plant) or collecting plant tissue somewhat haphazardly without much standardization among plant samples. Then samples are ground prior to collection of a standardized, homogenized subsample (e.g.,^9,23^). We will refer to this method as “*homogenizing tissue before subsampling”* (Fig. 1B). A criticism of this method is that differences in initial sample size are usually not accounted for (e.g., variation in total leaf area or root length). In this case, the size of the homogenized subsample is standardized, but not the amount of tissue *initially* homogenized. When the amount of plant tissue initially homogenized is inconsistent among samples, we do not know if the differences in microbial communities results from variation in the amount of plant material collected, or from true differences among the host plants. Differences in the detected species richness could thus be biased towards larger plant samples, or larger plants, rather than revealing true differences in richness and composition. Despite this criticism, the *homogenizing tissue before subsampling* method is relied on because it is thought that the homogenized pool from a relatively large initial sample may yield a more comprehensive subsample of the microbial community present within the plant. It is often assumed that *any* subsample from the powdered tissue, regardless of sampling approach used, will yield the same or a similar microbial community. To our knowledge, these assumptions have not been tested.

Because the optimal strategy may depend on the microorganism targeted, we tested these two strategies by extracting and amplifying bacterial DNA (16S rRNA gene), general fungal DNA (internal transcribed spacer region 2, hereafter ITS2) and arbuscular mycorrhizal fungal DNA (18S rRNA gene), from roots (all) and/or leaves (ITS2 only) of naturally occurring, mature, showy milkweed (*Asclepias speciosa*). We chose showy milkweed as a previous study has shown high colonization of both AM and general fungi^24^. Due to the highly diverse nature of foliar fungal endophytes (FFE) in general^14^, we extracted DNA from many additional subsamples per plant to assess the extent of undersampling within this group. Finally, we sampled leaves twice across the season to assess if broad-scale seasonal differences in FFE were still detectable despite potential undersampling, in order to better understand when—and for which type of questions—insufficient sampling is a problem. We predicted that (1) *homogenizing before subsampling* (strategy 2) would yield richer, more even microbial communities among plants, because more of the plant tissue would be initially homogenized, (2) different sampling strategies would result in different microbial community structures due to the spatial heterogeneity of microbes within plants, and (3) differences between sampling strategies would depend on the organism targeted and tissue sampled (roots or leaves), potentially due to inherent differences in global and likely local richness, as well as microbial modes of dispersal (above vs. belowground).

## Methods

### Field collections and sampling strategies

All collections occurred at the Teller Wildlife Refuge in western Montana, USA (46.3219 N 114.1292 W). The Teller Wildlife Refuge is a 525-ha property that comprises riparian, floodplain, wetland, and upland habitat. Showy milkweed grows in numerous patches across the property, concentrated near irrigation ditches, on the floodplain, and in old fields where sub-irrigation provides enough water. Plants were selected from a 500 m^2^ area of a field with a patchy distribution. All plants sampled were at least 3 m apart, and at each collection we sampled plants of similar size and developmental stage. During our first collection, we collected 6 individual leaves each from 20 showy milkweed plants on May 18th, 2016 before flowering, just as buds were beginning to form. We sampled from the same population again on September 6th, 2016 when flowers were present and seed pods were almost ripe. During the September collection we also collected as much of the root system as possible within the upper 20 cm of soil by destructively sampling each plant. Roots were washed with tap water to remove all visible soil, and fine roots (< 1 mm in diameter) were retained for further processing. All tissue was kept on ice upon collection, and then stored at − 20 °C. Bulk root collections weighed between 0.3 and 0.8 g dry weight, per plant. Bulk leaf collections weighed between 1.2 and 2.5 g dry weight per plant. To remove microbial tissues from root and leaf exterior and characterize only endophytic microbes, all root and leaf tissue was surface sterilized in 70% ethanol for 1 min., 0.5% NaOCl for 1 min. and then rinsed 2 × in sterile water. Imprints of sterilized tissue were made on growth medium to confirm the sterilization procedure. The absence of growth observed indicated successful surface sterilization. This process may not completely remove all non-viable surface DNA, however the combination of rinsing, and immersion of tissue in NaOCl and ethanol is effective at eliminating epiphyte tissue so that only residual DNA should remain^25^. After sterilization, leaves and roots from the September collection were separated into two groups based on the two sampling strategies we chose to compare.

For Strategy 1, *homogenizing after subsampling*, we removed one leaf disc from 6 leaves per plant (at random locations) using an 11.5 mm in diameter corkborer and placed the six discs per plant into single 1.2 ml Eppendorf tubes. We also used the same strategy for May leaf collections. This resulted in a total surface area of approximately 62 mm^2^ per plant, with a dry weight of approximately 30.0 mg of leaf tissue. For roots, approximately 30.0 mg of dry root segments per plant was placed into individual 1.2 ml Eppendorf tubes. As many DNA extraction kits recommend a maximum volume of approximately 20–30.0 mg dry weight or 50.0 mg fresh weight, we have found this to be a commonly used standard (e.g.,^26–30^), however, many studies lack sufficient detail on final extraction volumes. Subsampled root and leaf tissues were then freeze-dried using a Labconco Freezone benchtop freeze-dry system (Labconco, Kansas City, MO, USA). Subsampled tissue was homogenized using a 1600 MiniG tissue homogenizer and cell lyser (Spex SamplePrep, Metuchen, NJ, USA).

For Strategy 2, *homogenizing before subsampling,* all remaining root and leaf tissue from the September collection was subsequently freeze-dried and homogenized for each plant using the freeze-dry system and MiniG as described above. Approximately 30.0 mg dry weight per plant was then subsampled from the homogenized pool of root or leaf tissue and placed in 1.2 ml Eppendorf tubes for DNA extraction. All remaining foliar tissue from five plants collected in September (> 40 × more tissue per plant, equaling 1241–2467 mg per plant) was then divided into approximately 250 mg replicates for additional DNA extractions to assess the extent of undersampling.

### DNA extractions and PCR

DNA extractions of 20 foliar samples collected in May, as well as 40 foliar and 40 root samples representing the two different sampling strategies in roots and leaves in September (100 total extractions), were performed using the MO BIO PowerPlant Pro-htp DNA isolation kit, which has been shown to be comparable to other kits for extracting microbial DNA^31^. For five of the plants collected in September, we extracted DNA from all the remaining foliar tissue of the six leaves collected (1241–2467 mg dry weight per plant, compared to the 15–30 mg commonly used to infer differences in microbial community composition). This increased the amount of tissue analyzed per plant by > 40 ×. The number of additional extractions depended on the size of the leaves and ranged from 5 to 10 extractions per plant. These additional bulk material extractions on larger volumes were performed using the DNeasy Plant Maxi Kit (Qiagen, Hilden, Germany), also per manufacturer recommendations. To monitor potential background or cross contamination among samples, we also included control extractions and PCRs for all microbial groups and extraction kits used.

After extracting DNA, all samples were prepared for Illumina sequencing through a two-step PCR amplification. For root tissue we amplified the 18S rRNA gene, the internal transcribed spacer 2 (ITS2), and the 16S rRNA gene regions for AMF, general fungi, and bacteria respectively. In leaf tissue only the ITS2 general fungal region was targeted. Primer pairs for PCR1 included AMF specific primers WANDA^32^ and AML2^33^. For the ITS2 region we used a mixture of the fungal specific forward primers fITS7 and ITS7o^34,35^ and the general eukaryotic reverse primer ITS4^36^. AMF specific primers were used in addition to general fungal primers because ITS2 primers may not allow for a thorough characterization of AMF communities due to poor amplification of AMF when other fungi co-occur^37^. For the 16S rRNA gene, we targeted the V4 region using primers 515F and 806R^38^. More detailed descriptions of PCR reactions are in Bullington et al. (2018)^39^ and Lekberg et al. (2018)^37^. Briefly in PCR 1, each primer was flanked by 22 bp Fluidigm universal tags CS1 or CS2 (Fluidigm Inc. San Francisco, CA, USA). Each reaction contained a total volume of 12.5 μl which included 1.0 μl of DNA extract as template, 2.5 pmol of each primer in 1 × GoTaq Green Master Mix [(Green GoTaq Reaction Buffer, 200 μM dATP, 200 μM dGTP, 200 μM dCTP, 200 μM dTTP and 1.5 mM MgCl_2_) Promega, USA]. Reactions were performed in duplicate or triplicate, depending on concentration of amplified product (number of reactions was kept consistent within each microbial group), in a Techne TC-4000 thermocycler (Bibby Scientific, Burlington, USA) under the following conditions: initial denaturation at 95 °C for 2 min followed by 35 cycles at 95 °C for 1 min, 54 °C (18S rRNA gene), 57 °C (ITS2) or 50 °C (16S rRNA gene) for 1 min, and 72 °C for one min, with a final elongation for 10 min at 72 °C. To confirm the presence of our target amplicons, all reactions were analyzed by 1.5% agarose gel electrophoresis using a 100 bp ladder (GeneRuler DNA Ladder, Thermo Scientific, USA) as a size standard. As a precaution, control samples were sequenced even if they did not produce a band during gel electrophoresis.

All amplicons generated during PCR1 were diluted 1:10 for use as template in PCR2. PCR2 primer complexes consisted of the same Fluidigm tags (CS1 or CS2) as PCR1 primers, 8 bp Illumina Nextera barcodes (Illumina Inc., San Diego, CA, USA), and Illumina adapters. To minimize index hopping, we used unique dual indexing pooling combinations^40^, stored libraries individually, pooled only immediately before sequencing, and removed free adaptors from our libraries. PCR2 amplicons were purified using AMPure XP beads (Beckman Coulter Genomics, USA), quantified by Qubit 2.0 fluorometer (Invitrogen, USA) and pooled in equimolar concentration prior to sequencing. Sequencing was done at the Institute for Bioinformatics and Evolutionary Studies (IBEST) genomics resources core at the University of Idaho (http://www.ibest.uidaho.edu; Moscow, ID, USA). Amplicon libraries were sequenced using ¼ of a 2 × 300 paired-end (PE) run on an Illumina MiSeq sequencing platform (Illumina Inc., San Diego, CA, USA).

### Bioinformatics

Processing of raw sequence data was performed using “Quantitative Insights into Molecular Microbial Ecology 2" (QIIME2 version 2018.4; https://qiime2.org/)^18^. Sequence reads were first demultiplexed using the q2-demux plugin (https://github.com/qiime2/q2-demux). Only forward reads were used for the 18S rRNA gene region, as the overlap between forward and reverse reads is too short to merge the two without significant sequence loss. For the 18S rRNA gene only, forward reads were trimmed to 210 bp, which covers the informative region of our 18S rRNA gene target^33^. For ITS2 and the 16S rRNA gene, forward and reverse reads were trimmed where median quality score fell below 30, and if at any point quality score fell below 3 within the trimmed region, those sequences were removed from further analysis. The q2-dada2 plugin uses nucleotide quality scores to produce sequence variants (SVs), or sequence clusters with 100% similarity representing the estimated true biological variation within each sample. Although sequences are clustered at 100% similarity as opposed to the traditional 97% similarity, DADA2 produces fewer spurious sequences, fewer clusters, and results in a more accurate representation of the true biological variation present^41^. After DADA2 processing, microbial groups contained an average of 9000–17,000 sequences per sample (Supplementary Information, Table S1). All extraction and PCR controls were clean except for bacteria. Bacterial contaminants were subsequently removed from all samples to reduce potential background contamination. The database *MaarjAM*^42^ was used to assign taxonomy and remove non-target DNA for AMF, UNITE was used for general fungi (http://unite.ut.ee)^43^; and Greengenes was used for bacteria (http://greengenes.lbl.gov). All SVs that did not match with at least 70% identity (for bacteria) or 90% identity (for fungi) with at least 70% coverage to sequences within one of the above databases were removed. To help remove non-target DNA, we added many sequences representing non-target organisms (including many *Asclepias* spp.) to both our AMF and ITS databases to better identify contaminants and reduce misclassification when assigning taxonomy to these sequences. Taxonomy for each microbial group was then assigned using QIIME2 q2-feature-classifier (https://github.com/qiime2/q2-feature-classifier), a naive Bayes machine-learning classifier which has been shown to meet or exceed classification accuracy of existing methods^44^, setting a confidence threshold of 0.94 for fungi and 0.7 for bacteria. For 16S rRNA data, sequences identified as chloroplast or mitochondrial DNA were also removed, which resulted in the removal of > 90% of bacterial sequences (Supplementary Table S1). For non-AM root fungi, all Glomeromycota were removed in order to analyze AMF and non-AM root fungi separately.

### Statistical analyses

**Figure 2:**
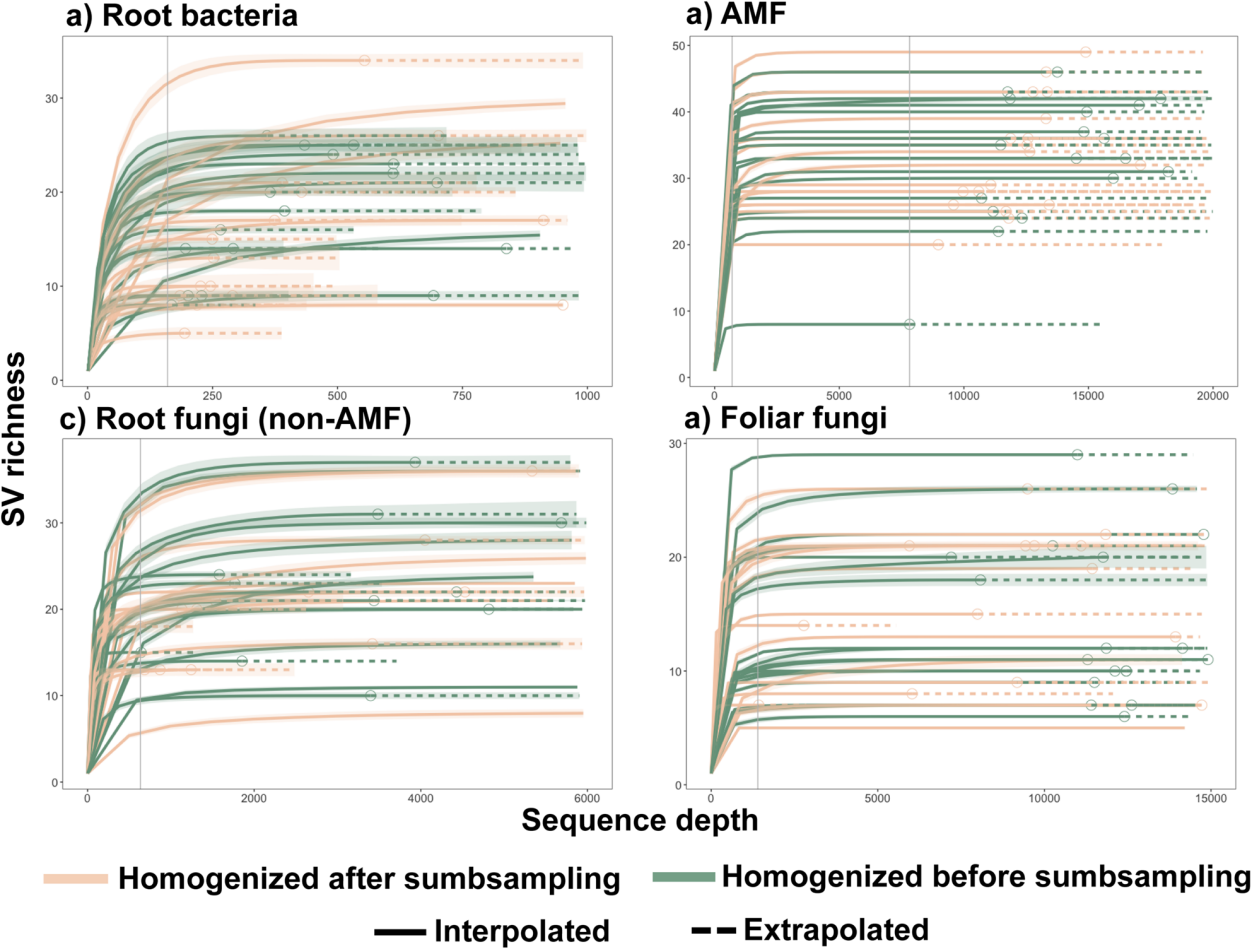
Sequence rarefaction curves for each sampling strategy (homogenizing after subsampling and homogenizing before subsampling) for (**a**) bacteria in roots, (**b**) Arbuscular mycorrhizal (AM) fungi in roots, (**c**) all non-AM fungi in roots and (**d**) foliar fungal endophytes, in *Asclepias speciosa* plants. Vertical lines represent the sequencing depth(s) at which each group was rarefied.

All statistical analyses associated with microbial community richness and composition in roots and shoots of *A. speciosa* were conducted in R^45^ using the vegan package^46^, except where otherwise noted. All analyses were based on rarefied data (160, 7820, 634 and 1400 sequences for bacteria, AMF, non-AM root fungi and FFE, respectively) using the ‘rrarefy’ function. Samples with few sequences were removed from further analyses to allow for greater sequencing depth, which resulted in 36, 36, 34 and 36 samples for bacteria, AMF, non-AM root fungi and FFE, respectively. Analyses of ITS2 data when extracting DNA from all remaining foliar tissue was based on a rarefaction level of 8475 sequences. These sampling depths were chosen based on saturation of sequencing effort curves of all reads after the removal of non-target DNA (Fig. 2) and in effort to retain the most samples (n = 18 per sampling strategy for bacteria, AMF and foliar fungi and 17 for non-AM root fungi). Sequencing effort curves were produced using the iNEXT package^47^. To test how sampling strategy influenced microbial alpha diversity metrics, we calculated richness as the number of SVs in each sample as well as Pielou’s ‘J’ evenness, which describes the similarity of species frequencies. To compare diversity metrics (based on SVs) between the two sampling strategies we performed a Wilcoxon signed-rank test on all paired values. To ensure that potential differences in sampling strategies were not due to artifactual SVs, we also compared results when implementing LULU, an algorithm for post-clustering curation that clustered SVs at 98.5% similarity and a minimum relative co-occurrence of 0.9^48^.

We performed non-metric multidimensional scaling (NMDS) to evaluate community structures for each sampling strategy and target region, individually. Each NMDS analysis was performed using the ‘metaMDS’ function and stress for all plots was between 0.04 and 0.16. These analyses were performed on Bray–Curtis distances of Hellinger transformed sequence abundances as well as Raup–Crick distances of presence/absence data. The ‘Procrustes’ function in vegan was used to assess similarity of patterns produced in the NMDS analyses for the paired sampling strategies and congruency was visualized in Procrustes plots. The ‘protest’ function was used with 1000 permutations to estimate the significance of the Procrustes statistic. We performed analyses on both presence/absence and abundance data to determine how low-abundant SVs influenced the differences between sampling strategies.

**Figure 3:**
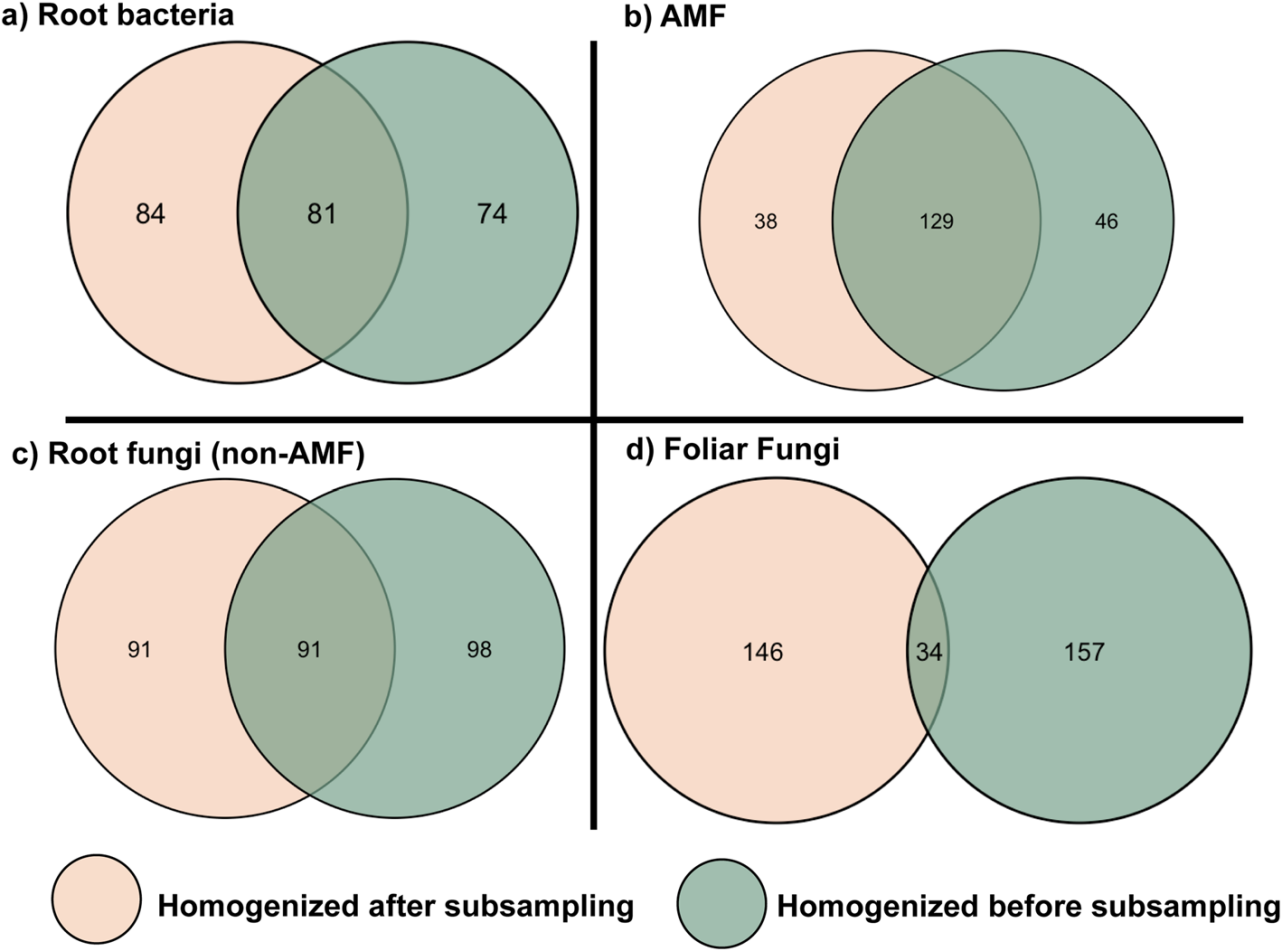
Venn diagrams showing the overlap of sequence variants (SVs) recovered from milkweed plants at a single site, when subsampling microbial communities using two different strategies.

**Figure 4:**
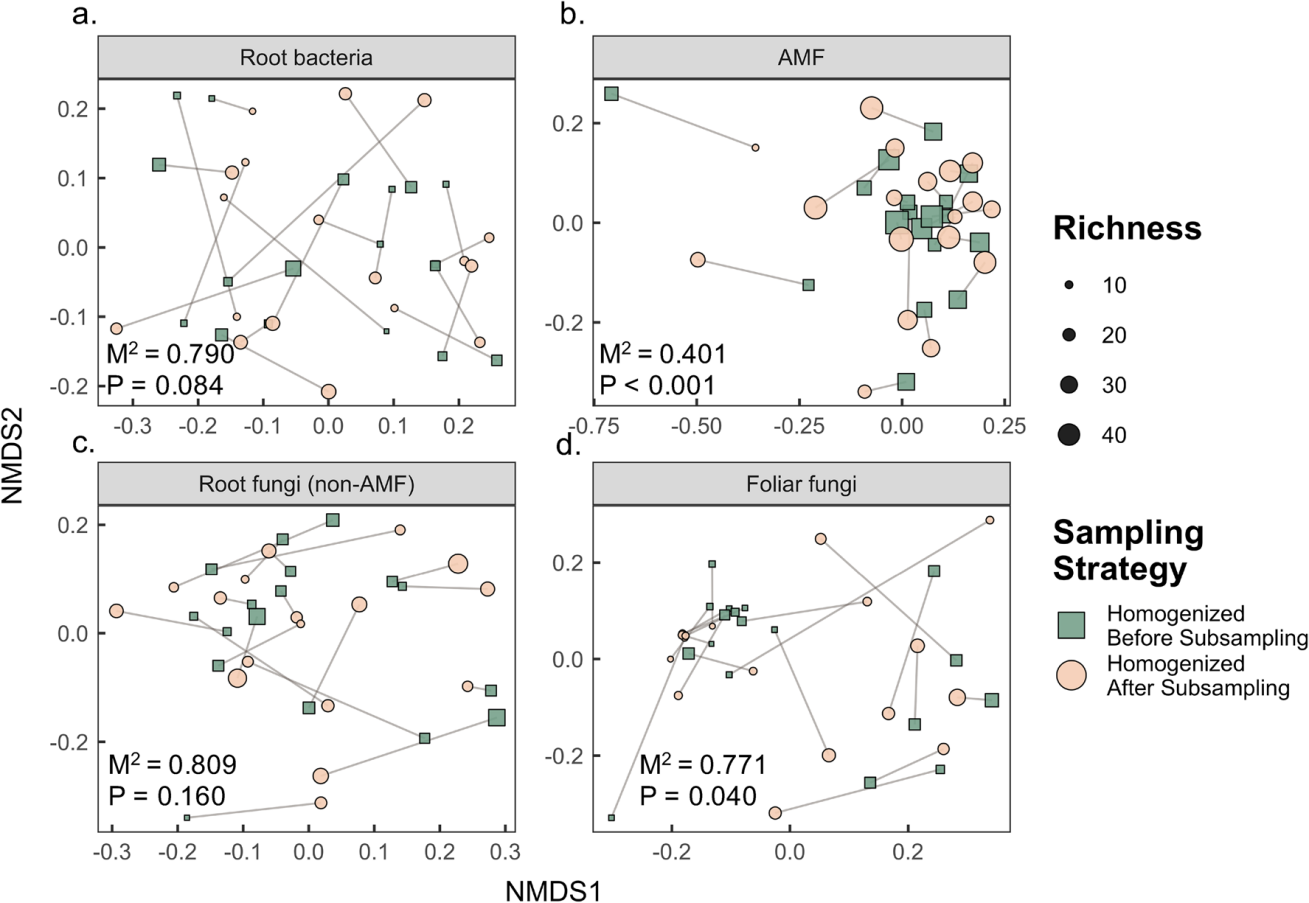
Procrustes plots comparing two different subsampling strategies used to characterize (**a**) root bacteria, (**b**) AMF, (**c**) non-AM root fungi, and (**d**) foliar fungi colonizing milkweed plants. Plots are based on non-metric multidimensional scaling of Bray–Curtis distance matrices of Hellinger transformed relative abundances. M^2^ values represent the sum of squared deviations between sample pairs, where lower values indicate a better fit between matrices.

**Figure 5:**
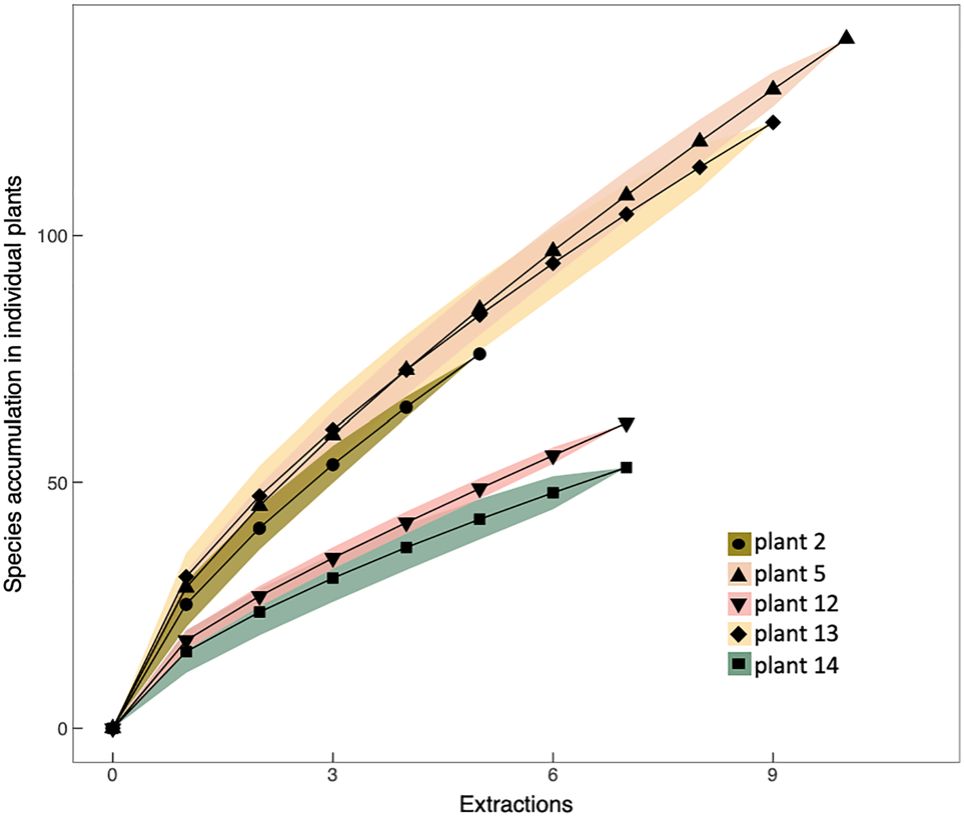
Species accumulation curves for foliar fungal endophytes colonizing milkweed (*Asclepias speciosa*). Each curve represents the SVs recovered from a single plant through additional extractions of bulk material (6 leaves per plant divided into ~ 250 mg extractions).

**Figure 6:**
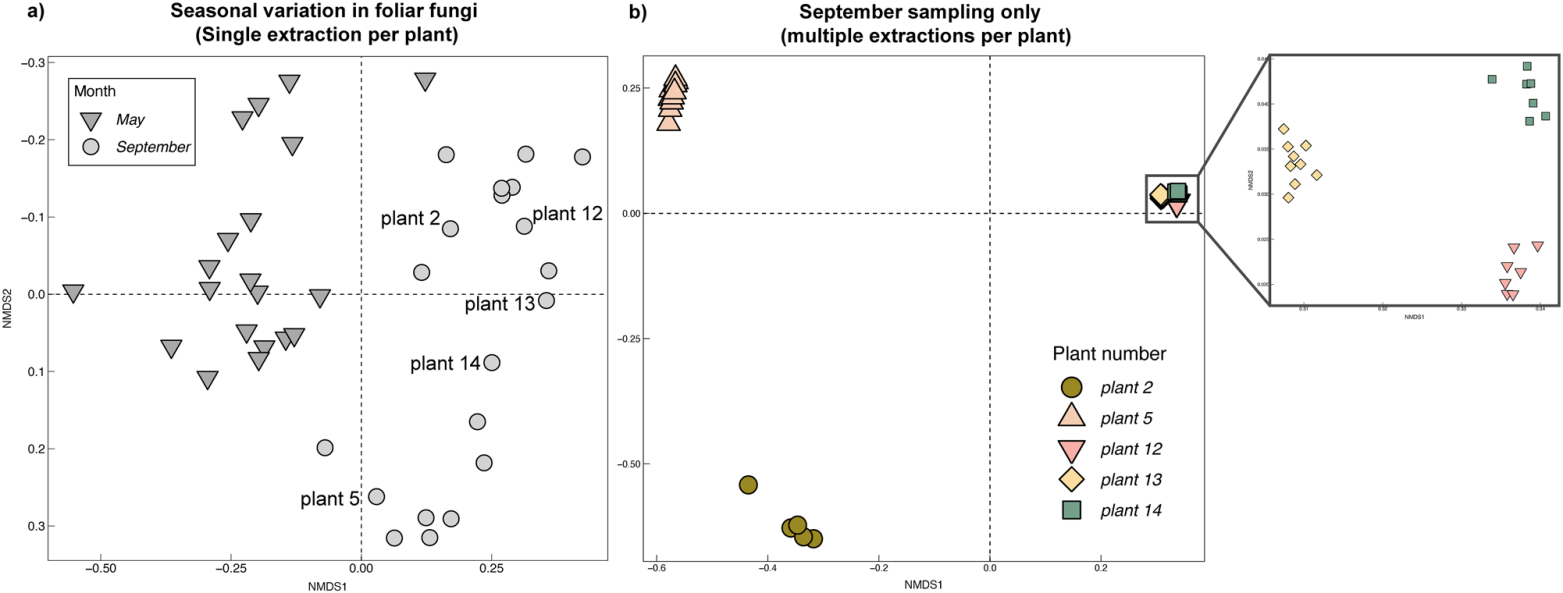
Nonmetric multi-dimensional scaling based on Bray–Curtis distances of foliar fungal endophytes in milkweed plants. Panel (**a**) represents the seasonal variation in foliar endophytic fungi when analyzing just one extraction rep per plant (leaf discs). Panel (**b**) represents the variation among plants when analyzing multiple extractions from each plant in September. The zoomed in panel highlights the less pronounced, but significant clustering among individual plants 2, 5, 12, 13 and 14.

To determine variation in microbial communities among individual plants when we extracted from multiple subsamples, we performed NMDS analyses as well as a PERMANOVA using the ‘adonis’ function. We also performed a PERMANOVA to detect seasonal differences between May and September leaf-disc collections. Figures 2, 3, 4, 5, 6 were generated using ggplot2^49^.

## Results

### Richness and evenness between sampling strategies

Contrary to our predictions, richness differed only for root bacteria where homogenizing before subsampling resulted in more SVs recovered than homogenizing after subsampling (p = 0.04, Supplementary Table S2and Fig. S1). Evenness did not differ for any microbial group. In addition, neither sampling strategy produced saturated species accumulation curves for any microbial community sampled, although AMF sampling approached saturation (Supplementary Fig. S2). This indicated inadequate sampling to characterize site richness, due to species turnover among plants. However, sequencing effort curves did saturate for all groups, indicating that sequencing depth was not a limiting factor in estimating the richness in individual samples (Fig. 2).

The most abundant SVs, (those occurring in at least half of our samples and recovered by both sampling strategies), represented only a small proportion of the microbial communities recovered. This included 5.5% of all AMF SVs (within the genera *Glomus* and *Clairoideoglomus*), while just 0.7% of total bacterial SVs (*Bacillus,* unknown bacteria) and 0.9% non-AM fungal SVs (*Nectriaceae, Plectosphaerella, Tetracladium*), fit these criteria. Foliar fungi were the most SV-rich group (Fig. 3), and only 0.8% of total SVs (*Mycosphaerella*) occurred in at least half of all samples and were recovered by both sampling strategies.

### Community overlap between sampling strategies

We examined the community overlap, that is, the total number and identity of individual SVs irrespective of their relative abundance, that were recovered from the milkweed population using both sampling strategies. AMF had the lowest total SV richness and the highest community overlap at 61% (Fig. 3). Even when rarefying AMF at 700 SVs as opposed to 7820 SVs, to more closely match rarefaction levels of other groups, overlap was maintained at 61% between sampling strategies. This indicated that it was not the higher rarefaction numbers that caused the greater overlap observed in this group. Sequencing rarefaction curves also showed sufficient sequence numbers to capture a majority of sample richness, even at the lower rarefaction levels. Bacteria and non-AM fungi in roots had a moderate overlap (34% each) and foliar fungal endophytes had the highest SV richness and the least overlap with just 10% of total SVs recovered by both sampling strategies.

To explore the extent to which low-abundant SVs influenced the differences between each sampling strategy, we gradually removed SVs that were represented by < 0.01% and then < 0.05% of total sequences (Supplementary Table S1). This resulted in the removal of SVs represented by fewer than 16, 2, 40, and 47 sequences (< 0.01%); and 78, 11, 201, and 235 sequences (< 0.05), for non-AM root fungi, bacteria, FFE and AMF, respectively. At each removal step, the overlap of microbial communities gradually increased (Table 1). After removing SVs that were represented by < 0.05% of all sequences, the AMF community overlapped 92% between the two sampling strategies. Non-AM fungi in roots overlapped 63%. Bacterial and FFE SV composition, however, remained more different than alike with just a 47% and 35% overlap, respectively. Since foliar fungi showed the greatest differences, we then compared SV overlap after clustering at 98.5% similarity and 90% co-occurrence. This coarser clustering did not increase the overlap between sampling strategies (7% as opposed to 10%), suggesting that at least for this group, the fact that we chose to cluster at 100% similarity using DADA2 did not explain the general lack of overlap.

**Table 1:** Total number and overlap of SVs when gradually filtering low abundant SVs of microbial communities recovered from milkweed leaves and roots using two different subsampling strategies (subsampling before homogenization and subsampling after homogenization of tissue).

SV overlap between sampling strategies				
	AMF	Root bacteria	Non-AM root fungi	Foliar fungi
Total SVs	213	239	280	337
% overlap of total SVs	60.6	33.8	33.9	10.1
Total SVs represented by > 0.01% of sequences	176	236	219	220
% Overlap of SVs represented by > 0.01% of sequences	72.7	34.5	40.5	15.4
Total SVs represented by > 0.05% of sequences	121	158	123	94
% Overlap of SVs represented by > 0.05% of sequences	91.7	46.8	63.4	34.5

Within individual plants, the number of SVs overlapping in both sampling strategies was highly variable, particularly for all non-AM fungal communities (Table 2). For foliar fungal endophytes, the percentage SV overlap within a single plant ranged between 0 and 36%. Similarly, for root bacteria, the percentage SV overlap ranged from 0 to 33% among individual plants. Three *A. speciosa* individuals yielded completely different communities between sampling strategies (0% overlap in SVs) for foliar fungi. Root bacteria and non-AM root fungi each had one plant with non-overlapping communities between sampling strategies (Supplementary Fig. S3). Three and four additional plants yielded just a single common SV between the two sampling strategies for FFE and non-AM fungi, respectively, whereas 2 plants had only 1 overlapping SV in root bacteria.

**Table 2:** Mean and standard deviation of the percent of bacterial and fungal sequence variants (SVs) recovered from individual milkweed plants using two different subsampling strategies (subsampling before homogenization and subsampling after homogenization of tissue).

Percentage of SVs recovered in individual *A. speciosa* plants				
	Root bacteria	AMF	Foliar fungi	Non-AM root fungi
Both methods	11.2% (4.6)	41.9% (9.7)	14.4% (14.3)	11.9% (10.0)
Homogenized after subsampling	40.1% (8.7)	29.5% (13.7)	40.7% (15.7)	45.1% (11.8)
Homogenized before subsampling	48.6% (8.6)	28.6% (10.1)	44.8% (15.2)	42.9% (15.5)

### Procrustes analyses reveal structural differences between sampling strategies

When we ran Procrustes analyses on bacterial and fungal groups using presence/absence data, we saw significant differences between sampling strategies for all groups except for AMF (Table 3). When considering the relative abundances of all SVs, as opposed to basing analyses on presence/absence, as we did above, the two sampling strategies still recovered significantly different microbial communities in root bacteria and in non-AM root fungi (Fig. 4, Table 3). For FFE, the two sampling strategies produced slightly more correlated community structures, but the similarity was weak (Table 3). This is likely because some plants had very similar FFE communities, while others had completely different FFE communities (Supplementary Fig. S3). Only AMF communities were consistently similar between sampling strategies when considering SV abundance.

**Table 3:** Results of Procrustes analyses comparing two sampling strategies used to sample microorganisms colonizing *A. speciosa* roots and foliar tissue. Procrustes analyses were performed on Bray–Curtis distances of Hellinger transformed SV (sequence variant) relative abundances as well as on Raup–Crick distances of presence/absence data. Significant values are highlighted in bold. M^2^ values represent the sum of squared deviations between sample pairs, where lower values mean a better fit between matrices.

Procrustes						
	Bray–Curtis (weighted)			Raup–Crick (presence/absence)		
	M^2^	PROTEST (r)	*p*	M^2^	PROTEST (r)	*p*
Root bacteria	0.790	0.458	0.084	0.788	0.461	0.094
AMF	0.401	0.774	**< 0.001**	0.351	0.806	**< 0.001**
Non-AM root fungi	0.809	0.437	0.160	0.855	0.380	0.393
Foliar fungi	0.771	0.478	**0.040**	0.852	0.384	0.144

### Multiple extractions per plant revealed severe undersampling of FFE

From the six leaves collected from plants in September 2016, we extracted DNA from all remaining leaf tissue (1240–2460 mg per plant). Despite increasing the processed plant tissue by two orders of magnitude, species accumulation curves for individual plants still failed to approach a plateau (Fig. 5). This was true even when clustering SVs at 98.5% using the LULU method. Post-clustering curation using the LULU method removed fewer than 10% of SVs and did not change the overall results. Where extractions from subsamples of 30 mg of leaf tissue (6 discs from 6 different leaves) had recovered 7–26 total foliar fungal SVs per plant, extracting from tissue representing our entire bulk collection of 6 leaves per plant or 10 × as much leaf tissue, resulted in 76–142 total SVs per plant. The average richness recovered by each extraction was 24 ± 8 SVs and the average number of unique SVs added by each consecutive extraction was 12 ± 6 SVs. Extractions from the same plant overlapped as little as 21% of SVs, showing that extractions from the same homogenized plant tissue can vary substantially, even when extracting from much larger volumes. Perhaps not surprisingly, larger leaves contained more FFE SVs than did smaller leaves, indicating that overall richness increases with plant size.

### Seasonal variation visible despite undersampling, but subtler differences were masked

With 30 mg subsampled per plant we were able to see broad seasonal differences between May and September (Fig. 6A). However, this approach did not allow us to differentiate among plants that harbored more similar communities. This became apparent when analyzing multiple replicates per plant (Fig. 6B). When we visualized the communities recovered in each additional extraction, we observed significant variation among plants that was not apparent when we only had community data from single 30 mg extractions. With the additional extractions we observed strong host filtering among individual plants (PERMANOVA, R^2^ = 0.91, P < 0.001). Subsequent pairwise analyses revealed that the fungal community within individual plants varied significantly from all other plants (Pairwise PERMANOVA, P < 0.01), and that three of the plants appeared much more similar to each other than the other two plants, potentially due to factors associated with their spatial distribution within the site.

## Discussion

### Different sampling strategies yield different microbial communities

The sampling strategies compared in this study (*homogenizing tissue before subsampling* and *homogenizing tissue after subsampling*) are common methods found in the literature for characterizing plant-associated microbial communities^14,23,26,29^. Procrustes analyses and community overlap between sampling strategies demonstrated that different strategies can capture disparate microbial communities within plants, with the extent of these differences depending on the community targeted and plant tissue type sampled. In FFE as well as bacterial and non-AM fungal communities in roots, subsamples from the same plant resulted in completely different sets of species recovered, illustrating the severe undersampling that is inherent to each of these strategies. With these sampling strategies, we are undoubtedly sacrificing power and accuracy to characterize the subtler aspects of plant microbiome interactions, despite often seeing community differences across landscapes, treatments or seasons.

Richness was higher when homogenizing before subsampling for bacteria only, despite differences observed in composition for all groups. It is perhaps surprising that homogenizing plant tissues before subsampling did not recover more species than homogenizing after subsampling for fungi as well, because with the former approach, more plant tissue is initially represented. Indeed, a previous study showed that sample pooling or homogenizing before subsampling resulted in a higher richness of soil fungi compared to equally sized individual samples^50^. In Song et al. (2015)^50^ they also found that multiple individual subsamples, rather than the single homogenized subsample, resulted in higher richness. This may suggest that the scale at which we are physically able to break down the particle size of plant tissues, as opposed to soil, is not always fine enough to sufficiently homogenize the fungi within. Because of this, plant-associated microbial communities may require a greater sampling effort than soil microbes. Additionally, the removal of low-abundant SVs did not result in differences in richness between the two sampling strategies for any microbial group, suggesting that neither strategy is better at capturing rare species. Although this study was performed only on milkweed plants, we believe that these results are applicable to other plant species as well. The richness reported here is similar to other studies of plant-associated microbes (e.g.^51,52^), indicating that differences in subsamples were not due to extreme richness of milkweed-associated microbes.

### Microbial diversity should inform sampling effort

The higher congruency that we saw between sampling strategies for AMF compared to other microbial communities may be due to the differences in their local and global estimated richness. While the global number of AMF species has been estimated in the hundreds to low thousands^42,53^, global estimates of fungal species in general range in the millions^54,55^. A recent global estimate of bacterial richness suggests similar scales^56^. In this study specifically, AMF had the lowest total SV richness and the greatest similarity between sampling strategies, while foliar fungal endophytes had the highest SV richness, and the lowest overlap of SVs between strategies. Since the amount of tissue sampled was equal for all microbial communities, the sampling effort was likely much higher for AMF (relative to the whole AMF community), than it was for bacteria and non-AM fungi. Consequently, with each sample we are likely sampling a much larger proportion of true AMF species richness.

Even though the estimated total community richness was highest for foliar fungi, the average estimated richness per individual plant was highest for AMF. This suggests that similar AMF SVs re-occurred across all plants with low species turnover. On the other hand, fungi in leaves had lower average richness per plant (Fig. 4, Supplementary Fig. S3), but the highest total richness, meaning that there was higher turnover of FFE species among plants sampled. These results may be a direct reflection of the overall community richness of the different microbial groups as well as their ability to spread and co-occur within plants. Based on these patterns, more individual plants and a greater sampling effort within individuals are likely needed to characterize FFE communities compared to AMF communities.

### Rare SVs contribute to variation among subsamples

Our results show that low abundant, rare SVs largely contributed to the differences seen between sampling strategies. Even AMF communities, which were already similar, increased in overlap by 50% between strategies after low abundant SVs (represented by < 0.05% of sequences, Table 1) were removed. Microbial community distributions are often characterized by long tails of low-abundant species^15^, and as such, the likelihood of resampling rare species in each replicate can be low. In one study, Zhou et al. (2011)^57^ randomly sampled a simulated community with an exponential distribution. They observed only a 53% overlap between two samples when sampling just 1% of that community. We see even more extreme differences in overlap in this study, where initial sampling effort is also low relative to the whole microbial community.

The importance of rare microbes may vary and is easily overlooked in favor of highly abundant, and perhaps more influential fungi or bacteria. However, due to the compositional nature inherent to amplicon data, those SVs that *appear* to be in low abundance at the time of sampling may only be relatively so. Also, we do not yet fully understand microbial species turnover or succession. Plant-associated microbial communities can change significantly in just a matter of months^58^, or even weeks^59^. In addition, the exact relationship between sequence number and biomass of a species is variable^60^, and there is little evidence, if any, that sequence number is in direct proportion with a species’ impact in an ecosystem. Some microbes may be more metabolically active than others, despite appearing to be present in smaller quantities^61^. The recovery of the rare microbial community is arguably just as vital as the recovery of species that appear more abundant.

Bioinformatics pipelines that artificially inflate the number of SVs, especially low abundant or rare SVs, could potentially inflate the differences we see among community subsamples. Hundreds of bioinformatics approaches have been used to analyze amplicon data, and no consensus exists on which is best. However, a recent study comparing the performance of 360 different software and parameter combinations showed that DADA2 (which is what we used here), with no other filter other than the removal of low quality and chimeric sequences, was best for recovering true richness and composition from a mock fungal community of 189 different strains^62^. If anything, DADA2 can erroneously lump closely related species^41^, which would make it more conservative than other methods used. However, in an effort not to overestimate the true variation between strategies compared in this study, we assessed the relative importance of rare taxa through the gradual removal of lesser-abundant sequences, and we also used LULU, which is sometimes employed to reduce artifactual diversity^48^. We also removed all SVs that could not be confidently assigned to known microbial taxa. Even with these approaches, substantial variation remained due to the inherent undersampling of the strategies compared.

### Severe undersampling obscures subtle community variation

With the possible exception of AMF, none of the sampling effort curves approached an asymptote meaning that both sampling strategies failed to adequately characterize the microbial communities present within a single plant or plant community (Supplementary Fig. S2). We found that multiple replicates from a single plant can vary by nearly 80% in FFE SVs, even when extracting from larger amounts (250 mg vs. 30 mg) of tissue. Lindahl et al*.* (2013)^63^ suggests that if duplicate subsamples differ much in community composition then these differences threaten to obscure finer-scale treatment effects and ecological correlations, and that sampling effort should be increased. Indeed, a more robust sampling effort through the use of multiple technical replicates revealed remarkably strong (R^2^ = 0.91), and significant host filtering within each individual plant that would have gone unobserved if extracting DNA from just a single replicate per plant. Although we may be able to observe patterns in under sampled data among sites or treatments, it is difficult to train models and make predictions or inferences in regard to the larger microbial population.

As Unterseher et al. (2011)^15^ suggests, it is often unnecessary to saturate richness in microbial communities, but this should be carefully considered before developing experiments and testing hypotheses. One must take into account the objectives of the study and the accuracy and precision required to meet those objectives. Although the methods traditionally employed to sample plant-associated microbes may be sufficient to generally observe landscape-scale differences, it is important to recognize that we are not characterizing these communities, rather we are taking a sliver of a ‘snapshot’ of species composition from a single point in time. A large proportion of true microbial diversity for most systems will likely still remain undetected and the specific results may be limited in their replicability.

### Summary and recommendations

Although it used to be common practice, multiple studies now suggest that duplicate and triplicate PCR reactions are unnecessary for fungi and bacteria^64,65^. However, based on the results of this study, we recommend the inclusion of a different kind of technical replicate (i.e., multiple extraction reps from a single plant), in addition to biological replicates (multiple plants in a single population), especially when studying factors that may generate subtler differences in plant associated microbial communities. We show that extracting DNA from the standard 25–30 mg (dry weight) per plant can result in microbial communities that vary by as much as 100% and extractions of 250 mg from a single plant can vary by as much as 79%. The need for increased replication is particularly important if site, treatment, or seasonal differences may be obscured by other environmental drivers. Striving for a more comprehensive understanding of the depth and structure of plant microbiomes and their response to their surrounding environments will help us to better understand the exact functions of plant–microbe associations and how we might manipulate plant microbiomes in order to reduce disease or increase plant productivity in the future.

Sample size and sampling effort have surpassed sequencing depth and cultivation as the bottleneck when characterizing plant-associated microbial communities. A good sampling design is essential to approximate underlying patterns in microbial community composition in a reproducible manner and both sampling effort and size should be clearly justified. Schloss (2018)^20^ elaborates on the concern of replicability and reproducibility with the growing use of Illumina-based studies of microbial communities, and describes PCR bias, sequencing errors, and cryptic or poorly described bioinformatics as preventing data from being generalizable to other environments. Undersampling and poor to absent descriptions of sampling effort and strategy also contribute to this problem, and the current frequency of undersampling should be concerning. The differences we see here between sampling strategies and the extreme variation among replicates suggest that many studies of plant-associated microbial communities may not be sufficiently replicable or reproducible.

Due to variation in community structure among AMF, bacteria, and non-AM fungi, standardizing a sampling protocol for all organisms is difficult, and best practices will, to some degree, depend on the specific organism targeted, richness, and site. Since neither sampling approach appeared to outperform the other, in many studies the overall sampling effort may be of greater importance. For example, when investigating landscape-scale differences in abundant or species poor microorganisms, a smaller sampling effort is often sufficient. However, we suggest that more diverse plant-associated microbial communities, such as foliar fungal endophytes and root-associated bacteria, necessitate a more robust sampling effort than what is currently practiced in the literature. Per sample richness, relative to the estimated total community richness should always be considered when determining the optimum sampling strategy for any system. For example, sampling strategies and volumes sufficient for sampling AMF communities in extreme environments are likely not adequate for sampling fungal endophytes in the tropics where richness is high^66^. The need for increased sampling effort is especially pertinent if noise associated with sites, treatments or sample processing may potentially obscure the differences among them. In studies that fail to see differences in microbial communities among sites or treatments, sampling effort should always be examined as a potential impediment.

In summary, we recommend that: (1) authors provide more transparent, detailed sampling information as well as sequencing and sampling effort curves, (2) sampling effort is not arbitrary, but is adjusted based on the diversity of plant microbiomes and per sample richness relative to total community richness (both of which may require preliminary sampling), and (3) authors consider increased sampling effort when investigating smaller-scale drivers of microbial communities such as host filtering or subtle gradients, or when attempting to truly characterize microbial communities. Finally, controlling for the amount of plant tissue sampled both *before* and *after* homogenization, although not tested here, may be the most optimal strategy for reducing potential bias. Standardizing how we sample plant-associated microbial communities as suggested by Dickie et al. (2018)^19^, or at the very least, insisting on more robust and transparent sampling strategies, will allow for more accurate and comprehensive analyses as well as better cross-study comparisons in the future.

## Supplementary Information

Supplementary Information.

## Data Availability

Raw amplicon sequence data: NCBI Sequence Read Archive (SRA), BioProject accession number PRJNA633878. Sequence variants: GenBank (https://www.ncbi.nlm.nih.gov/genbank) accessions: MN029770-MN030137 (FFE); MN029143-MN029446 (AMF); MN029447-MN029769 (root fungi); MN069335—MN069498 (root bacteria). Community matrices and metadata: Figshare https://doi.org/10.6084/m9.figshare.11741673.
