# Supplementary material for: Insufficient sampling constrains our characterization of plant microbiomes
Source: Sci Rep. 2021 Feb 11;11:3645. doi: 10.1038/s41598-021-83153-9 (PMC7878899; doi:10.1038/s41598-021-83153-9)
Supplement: Supplementary file 1 — Supplementary Information. [file 41598_2021_83153_MOESM1_ESM.docx]

**Supplementary Information for:**

**“Insufficient sampling constrains our characterization of plant microbiomes”**

Lorinda S. Bullington^1*^, Ylva Lekberg^1,2^, Beau G. Larkin^1^

^1^MPG Ranch Missoula, MT, 59801, USA, [lbullington@MPGRanch.com](mailto:lbullington@MPGRanch.com); ^2^Department of Ecosystem and Conservation Sciences, University of Montana, Missoula, MT, 59812, USA

**Table of Contents:**

| **Supplemental Table 1** | Page 2 |
| --- | --- |
| **Supplemental Table 2** | Page 3 |
| **Supplemental Figure 1** | Page 4 |
| **Supplemental Figure 2** | Page 5 |
| **Supplemental Figure 3** | Page 6 |

**Supplementary Table 1.**

Total number of sequences at each step in the bioinformatics pipeline, for all microbial groups.

**Supplementary Table 2. Spearman’s rank test comparing sequence variant richness and evenness between sampling strategies.**

| **Root Bacteria** | | |
| --- | --- | --- |
|  | SV richness | Evenness |
| Wilcoxon signed rank test (V, pval) | **32, 0.04** | 51, 0.14 |
|  |  |  |
| **AMF** | | |
|  | SV richness | Evenness |
| Wilcoxon signed rank test (V, pval) | 157, 0.89 | 177, 0.65 |
|  |  |  |
| **Root-Fungi (non-AMF)** | | |
|  | SV richness | Evenness |
| Wilcoxon signed rank test (V, pval) | 69, 0.74 | 96, 0.38 |
|  |  |  |
| **Foliar Fungi** | | |
|  | SV richness | Evenness |
| Wilcoxon signed rank test (V, pval) | 82, 0.81 | 70, 0.52 |

**Supplemental Figure 1**. Boxplots comparing richness and Peilou’s J evenness recovered by each sampling strategy for microbial communities colonizing root and leaf tissue of 17-20 *Asclepias speciosa* plants.

**Supplemental Figure 2.**

Species accumulation curves corresponding to two different sampling strategies (homogenizing before subsampling and homogenizing after subsampling) for microbial communities colonizing root and leaf tissue of *Asclepias speciosa* plants.

**Supplementary Figure 3.** Stacked bar chart depicting the overlap in sequence variants (SVs) per individual plant, for both sampling strategies for microbial communities colonizing root and leaf tissue of *Asclepias speciosa*.
